# Patient-Ventilator Synchronization During Non-invasive Ventilation: A Pilot Study of an Automated Analysis System

**DOI:** 10.3389/fmedt.2021.690442

**Published:** 2021-07-07

**Authors:** Christophe Letellier, Manel Lujan, Jean-Michel Arnal, Annalisa Carlucci, Michelle Chatwin, Begum Ergan, Mike Kampelmacher, Jan Hendrik Storre, Nicholas Hart, Jesus Gonzalez-Bermejo, Stefano Nava

**Affiliations:** ^1^Normandie Université — CORIA, Avenue de l'Université, Saint-Etienne du Rouvray, France; ^2^Servei de Pneumologia, Corporació Parc Taulí, Sabadell, Spain; ^3^Departament de Medicina, Universitat Autònoma de Bellaterra, Barcelona, Spain; ^4^Service de Réanimation Polyvalente, Unité de Ventilation à domicile, Hôpital Sainte Musse, Toulon, France; ^5^Pulmonary Rehabilitation, Istituti Clinici Scientifici Maugeri, Istituto di Ricovero e Cura a Carattere Scientifico, Pavia and Department of Medicine and Surgery, Respiratory Diseases, University of Insubria, Varese-Como, Italy; ^6^Clinical and Academic Department of Sleep and Breathing, Royal Brompton & Harefield, National Health Service Foundation Trust, London, United Kingdom; ^7^Division of Intensive Care, Department of Pulmonary and Critical Care, Faculty of Medicine, Dokuz Eylul University, Izmir, Turkey; ^8^Department of Pulmonology, Antwerp University Hospital and Antwerp University, Antwerp, Belgium; ^9^Department of Pneumology, University Medical Hospital, Freiburg, Germany; ^10^Pneumologie Solln, Munich, Germany; ^11^Lane Fox Clinical Respiratory Physiology Research Centre, Guy's and St Thomas' NHS Foundation Trust, London, United Kingdom; ^12^Sorbonne Université, INSERM, UMRS1158 Neurophysiologie Respiratoire Expérimentale et Clinique, Paris, France; ^13^AP-HP, Groupe Hospitalier Pitié-Salpêtrière Charles Foix, Service de Soins de Suites et réhabilitation respiratoire-Département R3S, Paris, France; ^14^Respiratory and Critical Care, Sant'Orsola Malpighi Hospital, Alma Mater Studiorum, University of Bologna, Department of Specialistic, Diagnostic and Experimental Medicine (DIMES), Bologna, Italy

**Keywords:** non-invasive ventilation, patient ventilator asynchrony, chronic obstructive pulmonary disease, ineffective triggering, monitoring, automatic scoring

## Abstract

**Background:** Patient-ventilator synchronization during non-invasive ventilation (NIV) can be assessed by visual inspection of flow and pressure waveforms but it remains time consuming and there is a large inter-rater variability, even among expert physicians. SyncSmart™ software developed by Breas Medical (Mölnycke, Sweden) provides an automatic detection and scoring of patient-ventilator asynchrony to help physicians in their daily clinical practice. This study was designed to assess performance of the automatic scoring by the SyncSmart software using expert clinicians as a reference in patient with chronic respiratory failure receiving NIV.

**Methods:** From nine patients, 20 min data sets were analyzed automatically by SyncSmart software and reviewed by nine expert physicians who were asked to score auto-triggering (AT), double-triggering (DT), and ineffective efforts (IE). The study procedure was similar to the one commonly used for validating the automatic sleep scoring technique. For each patient, the asynchrony index was computed by automatic scoring and each expert, respectively. Considering successively each expert scoring as a reference, sensitivity, specificity, positive predictive value (PPV), κ-coefficients, and agreement were calculated.

**Results:** The asynchrony index assessed by SynSmart was not significantly different from the one assessed by the experts (18.9 ± 17.7 vs. 12.8 ± 9.4, *p* = 0.19). When compared to an expert, the sensitivity and specificity provided by SyncSmart for DT, AT, and IE were significantly greater than those provided by an expert when compared to another expert.

**Conclusions:** SyncSmart software is able to score asynchrony events within the inter-rater variability. When the breathing frequency is not too high (<24), it therefore provides a reliable assessment of patient-ventilator asynchrony; AT is over detected otherwise.

## 1. Introduction

Nocturnal non-invasive ventilation (NIV) is recognized as an effective treatment for chronic hypercapnic respiratory failure. Monitoring NIV during sleep is a necessary adjunct to daytime assessment of the treatment, which may affect prognosis, quality of sleep, or morning dyspnea (1–4). A systematic approach for determining undesired events such as leaks, upper airway obstruction (UAO) with or without a decrease in respiratory drive, and patient-ventilator asynchrony (PVA) from polygraphy performed under NIV was provided by the SomnoNIV group (5–7). An asynchrony index (AI) greater than 10% is quite often observed (8–12) and may be sleep-dependent (13). PVA is often the source of discomfort (11, 14). During long-term home NIV, a high prevalence of ineffective efforts (IEs) in patients with obstructive and restrictive diseases using polygraphic assessment was reported by Fanfulla et al. (13) and Guo et al. (10) and was later confirmed by Ramsay et al. with a parasternal EMG (12).

In general, waveforms of pressure and flow contain all the necessary information required for identifying the type of asynchrony events (AEs) (6, 7, 9, 11, 15–19). Pressure waveforms provide a direct access to ventilator cycles. The flow results from a combination between ventilator cycles and patient breathing cycles. Interpreting these waveforms is not always simple, primarily because the interplay between these two cycles is not trivial. Indeed, the ability of intensive care unit (ICU) physicians to score AEs is quite low (20, 21) and scarcely implemented due to a lack of skills (22). Nevertheless, IEs can be reliably detected by visual inspection (19). Specific training in mechanical ventilation increases the ability to identify AEs from waveforms, but the years of experience are not necessarily associated with a better ability to recognize the three main AEs [IE, double-triggering (DT), and auto-triggering (AT), the latter one being less often well-recognized] (21).

An automatic analysis of the pressure and flow waveforms is required to substantially shorten the long duration spent performing visual scoring (23). It was shown that detection of IEs by the means of an algorithm applied to the flow and pressure in the ventilation circuit was possible (24–29). All studies, but two, were performed in invasive ventilation where the leak is not relevant. Moreover, either only IEs were often considered (24, 25, 27) or only AEs without any further specifications (26, 28, 30). DT has only been considered in two studies (29, 31). ATs were never investigated with an automatic detection. It is therefore important to develop an algorithm to automatically detect the three main AEs (IE, DT, and AT) for NIV. The SyncSmart™ software was developed for such purpose, which works from the pressure and airflow waveforms sampled at the device rate (here 64 Hz). This study aimed to is to assess the ability of this algorithm to detect the three main PVA events during pressure support ventilation (PSV) by only processing flow and pressure waveforms.

## 2. Materials and Methods

Nine stable patients monitored under NIV (PSV mode with a backup frequency) in the Pneumology Unit Care of the Corporació Parc Tauli (Sabadell, Spain) were included in this study. The data were randomly selected from a study focused on the prevalence of AEs. This study was approved by the local ethics committee of Corporació Parc Tauli (CIR2010/015). Written informed consent was obtained from patients. Nine expert physicians from France, Germany, Italy, The Netherlands, Spain, Turkey, and the United Kingdom reviewed the automatic scorings.

Two restrictive patients, one obstructive patient, three patients with chronic obstructive pulmonary disease (COPD), and three patients with amyotrophic lateral sclerosis (ALS) were selected. They were ventilated with different devices: three with a Vivo 40 (Beas Medical, Mölnlycke, Sweden), four with a Lumis 150, one with an Astral 150 (ResMed, North Ryde, Australia), and one with a Trilogy (Philips Respironics, Murrysville, USA). Each patient used a full-face mask. The waveforms were recorded in napping patients in their most comfortable position during the initiation of mechanical ventilation or during routine controls in a quiet room. Flow *Q*, airway pressure *P*_aw_, and belt waveforms *B*_thorax_ and *B*_abdom_ were measured. The data were acquired using an external polygraph (Powerlab 16Sp, ADInstruments, Sydney, Australia), equipped with a pressure transducer (model 1050, ADInstruments, Sydney, Australia) and a pneumotachograph (model S300, instrumental dead space 70 ml, resistance *r*_p_ = 0.0018 cmH_2_O/l/s, ADInstruments, Sydney, Australia), both inserted in the ventilation circuit close to the mask and with respiratory inductance plethysmography belts (Pro-Tech, Canada). The polygraph was connected to a computer equipped with data acquisition software (Chart 7.0, ADInstruments, Sydney, Australia). The sampling frequency of measurements was *f*_s_ = 1, 000 Hz, but the data were then resampled at the frequency *f*_V_ = 64 Hz, as used by SyncSmart software. The acquired data were read by the SyncSmart software in a text format.

**Figure 1:**
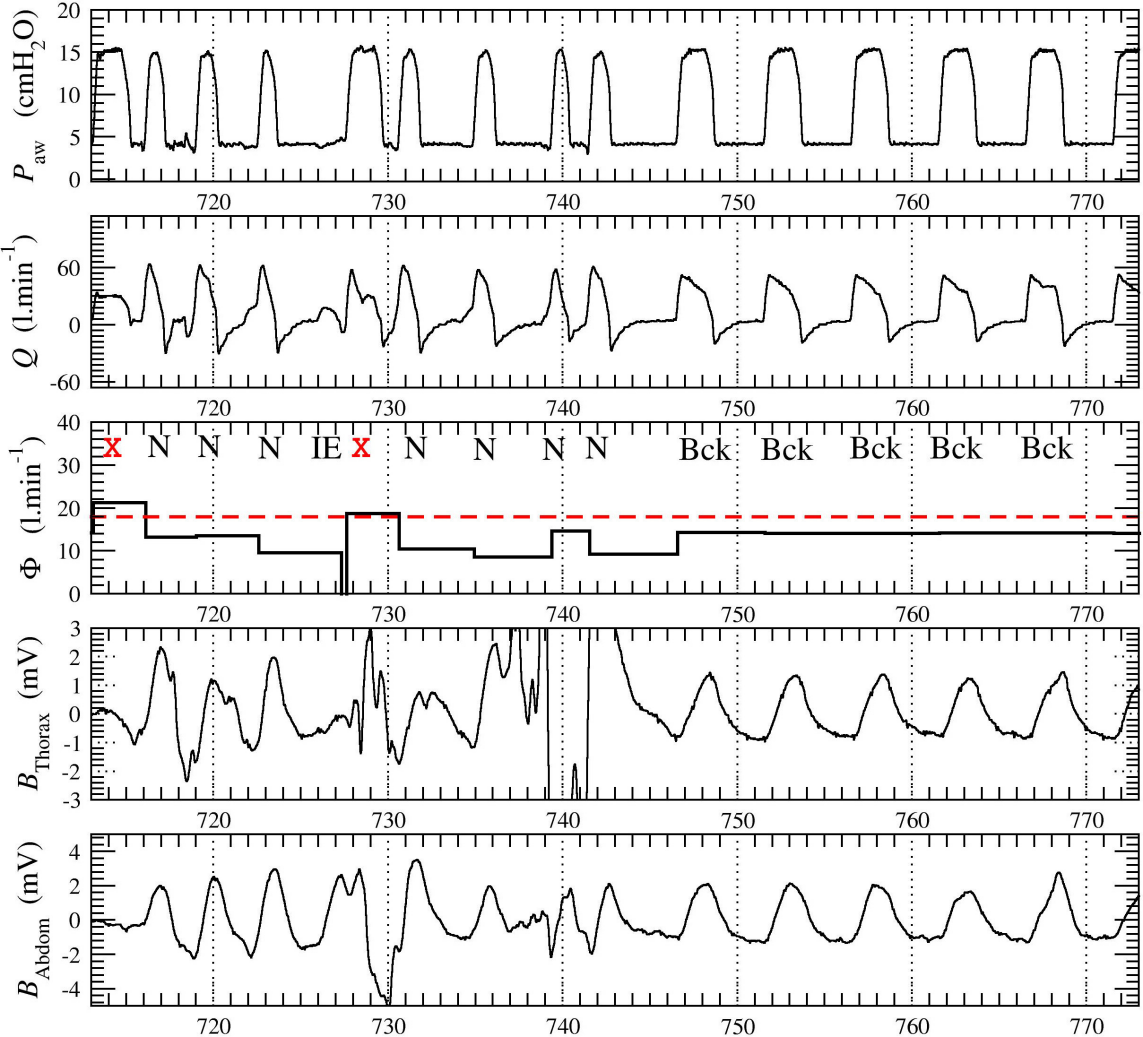
Excerpt from the 20-min times series recorded in patient 1. The leakage Φ is averaged over each ventilatory cycle. The belt signals *B*_thorax_ and *B*_abdom_ were provided to the physicians for reviewing the automatic scoring. The ventilatory cycles at *t* = 713 s and *t* = 728 s (marked with red cross) were automatically discarded by SyncSmart due to its excessively turbulent aspect and because the leakage Φ exceeds the threshold Φ_t_, here equal to 18 l min^−1^.

For each patient, we recorded a long session of NIV. One 20-min window of data was extracted from each of the recordings: The selected data window starts at least 5 min after the beginning of the session to avoid transient patient-ventilator interactions as commonly observed. No other criterion was used to select these windows. The belt signals, only used by the expert physicians to review the automatic scoring produced by the SyncSmart software, were filtered to remove the long-term drift and to improve their readability. A typical excerpt of the four measured waveforms with the total (intentional and non-intentional) leakage computed by SyncSmart is shown in Figure 1.

**Figure 2:**
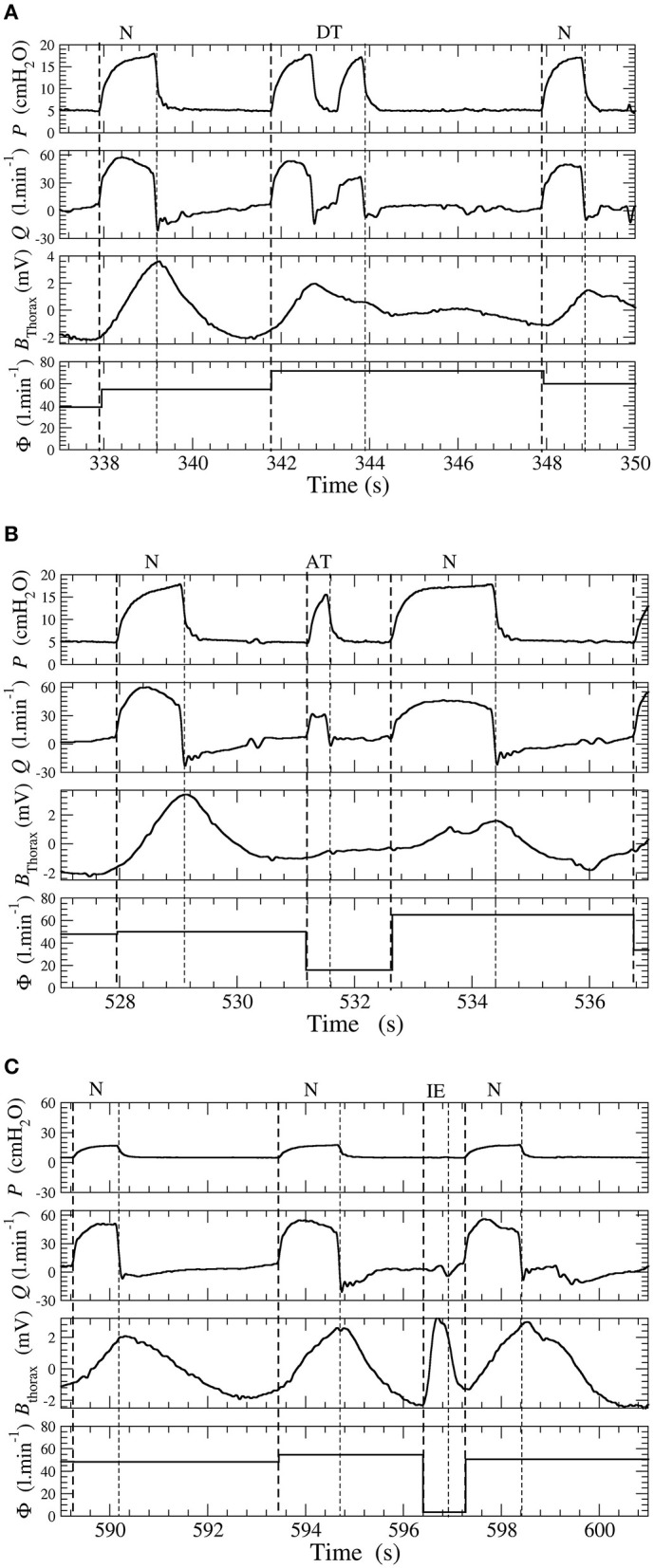
**(A)** Double-triggering. **(B)** Auto-triggering. **(C)** Ineffective effort. Examples of double-triggering (DT), auto-triggering (AT), and ineffective efforts (IEs) in a patient ventilated with a pressure support ventilation (PSV) mode. Waveforms of pressure *P*, flow *Q*, thoracic belt *B*_thorax_, and total leakage Φ.

The SyncSmart software is analyzes the pressure *P*_aw_ and the flow *Q*, either measured by the ventilator or by the external sensors as in the present protocol. The SyncSmart software considers three ventilator settings: expiratory positive airway pressure (EPAP), inspiratory positive airway pressure (IPAP), and the backup frequency (*f*_bck_) at which the ventilator delivers the pressure cycles. The software computes a leakage


(1) $$\Phi =\frac{1}{t_{\text{end}}-t_{\text{init}}}\int_{t_{\text{init}}}^{t_{\text{end}}}Q(t)\text{d}t$$

where *t*_init_ is the time at which the inspiratory, effort is initiated and *t*_end_ is the time at the end of expiration (*t*_end_ is also the time at which the next inspiratory effort is initiated). According to this equation, the leakage Φ has a constant value over each cycle. This leakage is used to discard the excessively turbulent parts of the waveforms. As exemplified in Figure 1, it is impossible to reliably score these cycles, neither by visual inspection by expert physicians nor by an automatic algorithm. The corresponding ventilatory cycles are thus marked with a red cross and are discarded from the statistical analysis. The events scored by SyncSmart software are shown in Figure 2 and are defined as follows.

DT Double-triggered ventilatory cycles for which there are two pressure rises during one inspiratory effort;
AT Auto-triggered ventilatory cycles when the pressure rise is not triggered by an inspiratory effort and thus occurs during the expiratory phase;
IE Ineffective efforts when there is a patient inspiratory effort, which is not followed by a pressure cycle delivered by the ventilator;
Bck Backup cycles when the pressure rise is triggered by the ventilator according to the backup frequency *f*_bck_;
N Ventilatory cycles are considered normal when there is absence of one of the events described above.

The asynchrony index (AI) is defined as


(2) $$\begin{array}{l} \text{AI}=100\frac{N_{\text{DT}}+N_{\text{AT}}+N_{\text{IE}}}{N_{\text{tot}}}; \end{array}$$

where *N*_tot_ is the number of ventilatory cycles (N, Bck, DT, AT, IE, and discarded cycles). AI is expressed as a percentage.

All the physicians have been working with NIV for 21.5 ± 7 years and, consequently, are considered as experts with NIV. All of them were asked to independently review the automatic scoring of PVA produced by SyncSmart according to their skills and knowledge. Expert physicians were reviewing the scoring by visual inspection of the pressure and flow as used by the SyncSmart software in addition to the belt signals as recommended by Gonzales-Bermejo et al. (6) and Longhini et al. (32), providing a reliable gold standard scoring. Since there is a long experience in sleep scoring for reviewing automatic scoring, we chose to reproduce the corresponding methodology: starting from the automatic scoring, we asked each expert physicians to individually correct it, and then, we compared the corrected scoring among each other and with the automatic scoring (33–35). The software contains a functionality that allows the physician to review the automatic scoring and to correct the type of ventilatory cycle. The physician may also add or remove a ventilatory cycle. Since the expert physicians have the belt signals, they can identify UAO which may be defined by the decrease in patient flow during a pressure cycle delivered by the ventilator (6). By definition, there is no effective IE and the pressure rises are triggered by the backup frequency *f*_bck_: The SyncSmart software thus scored these ventilatory cycles as Bck cycles. Consequently, backup cycles scored as obstruction by physicians are retained as Bck cycles for the statistical analysis.

Physicians were thus asked to focus on the main PVA events, that is, on IE, AT, and DT. Physicians were moderately under “time pressure” since they had approximately 2 min to review 1 min of tracing. Each physician reviewed at least 110 min of tracing; consequently, each patient recording was reviewed by at least five expert physicians. The automatic scoring was then compared to the reviewed scoring of each expert, which was considered as a reference. Inter-rater comparisons were also computed by selecting successively each scorer as a reference and then by computing the mean values. The number of each type of AE and the was computed. We computed the sensitivity (Se), specificity (Sp) PPV, κ-coefficients, and agreement when SyncSmart scorings were compared with the scorings of the experts. These quantities were also computed to compare experts with each other as commonly carried out for assessing inter-rater agreement in sleep scoring (33–35). Student's *t*-test is used with a significance level at 0.05.

## 3. Results

Characteristics of the patient at enrollment and ventilator settings are reported in Table 1. Five patients were well-established on mechanical ventilation, and four were recently initiated. A total of 4,201 ventilatory cycles were analyzed.

**Table 1:** Patient characteristics.

**#**	**G**	**BMI**	**Disorders**	***T*_T_**	**I_**PAP**_**	**E_**PAP**_**	***f*_bck_**	**Device**
1	F	26	Restrictive:	>48	16	4	12	Breas Vivo 40
			Kyphoscoliosis + hiatal hernia					
2	F	34	OHS	<2	13	3	14	ResMed Lumis 150
3	F	30	Restrictive:	<2	20	5	18	Breas Vivo 40
			kyphoscoliosis + Obesity					
4	F	37	COPD	>120	21	10	12	ResMed Lumis 150
5	M	28	COPD	24	21	5	10	ResMed Lumis 150
6	M	23	COPD	12	22	6	14	ResMed Lumis 150
7	F	22	ALS	1	13	4	16	Respironics Trilogy
8	F	78	ALS	3	20	6	12	ResMed Astral 150
9	F	22	ALS	9	17	4	14	Breas Vivo 40

*Experience of a patient in mechanical ventilation is reported in months. The ventilator type is also reported*.

*G, Gender; BMI, body mass index (kg.m^−2^); T_T_, training period in months; IPAP, inspiratory positive airway pressure (cmH_2_O); EPAP, expiratory positive airway pressure (cmH_2_O); f_bck_, backup frequency (cycle per min)*.

**Figure 3:**
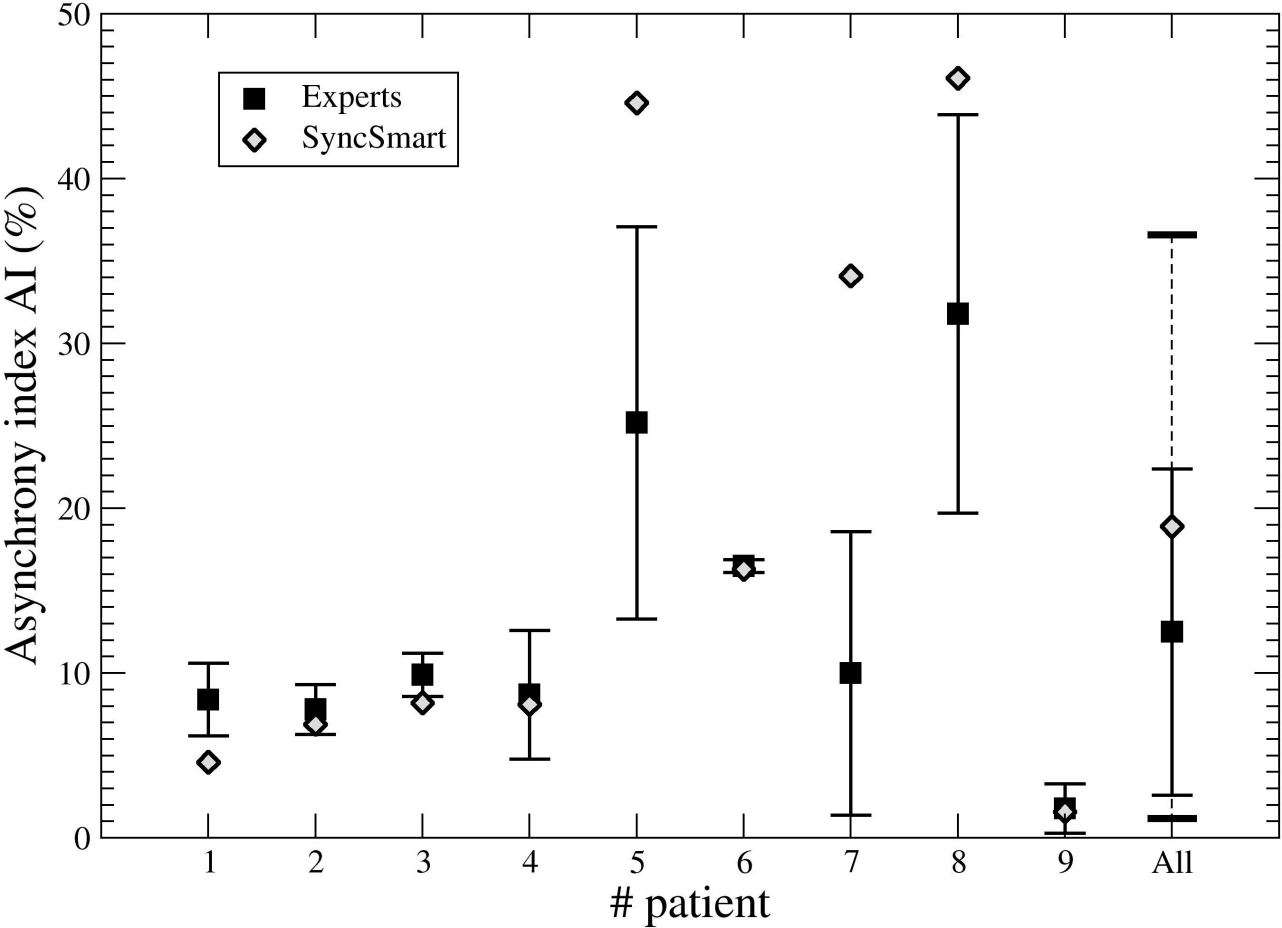
Asynchrony index (AI) computed from the SyncSmart scoring and the mean AI (with the standard deviation) computed from the expert scorings for each patient and for all patients, respectively.

The nine physicians performed the analysis of the flow, pressure, and belt waveforms using the SyncSmart graphical interface. The number of AEs as scored by SyncSmart and as scored by the experts is reported for each patient in Table 2. On average, the SyncSmart software reported the same smaller numbers of DT (4.1 ± 3.6*vs*. 6.6 ± 13.0, *p* = 0.31) and IE (24.1±21.7*vs*. 22.0±21.5, *p* = 0.39) as the by experts. Conversely, the automatic scoring reported significantly more AT than the experts (71.7±88.0 vs. 25.5±35.3, *p* = 0.0036). The Se, Sp, PPV, κ-coefficient, and agreement are reported in Table 3 in which the results provided by SyncSmart software are successively compared with each expert, and where each expert is compared to one another. The AI assessed by SyncSmart is not significantly different from the at assessed by the experts (18.9 ± 17.7% vs. 12.8 ± 9.4%, *p* = 0.19) as shown in Figure 3. Se and Sp provided by SyncSmart for N, Bck, DT, and IE are significantly greater than those provided by experts when compared to one another. The PPV for IE is significantly greater for the automatic scoring than for experts. The inter-scorer variability for DT and AT is nearly twice that of IE. AT is clearly the event that leads to the most important discrepancies between the automatic scoring and the experts (Table 2), particularly for patients 5, 7, and 8 (Table 4).

**Table 2:** Distribution of asynchrony events (AEs) according to the automatic scoring by SyncSmart.

		***N*_tot_**	***N*_IE_**	***N*_AT_**	***N*_DT_**	**AI**	***N*_Bck_**	***N*_X_**	***N*_mod_**	***f*_breath_**
1	S	324	5	7	3	4.6%	237	36	–	13
	E	307	9.4	14.1	1.6	8.4%	186		54	
			(4.0)	(7.0)	(1.2)	(2.2)	(27.8)		(12.6)	
2	S	375	16	6	4	6.9%	105	1	–	15
	E	360	22.0	3.5	2.7	7.8%	84.7		41.0	
			(4.9)	(2.2)	(1.5)	(1.5)	(9.3)		(15.8)	
3	S	565	41	0	5	8.2%	39	14	–	22
	E	541	46.5	5.2	4.0	10.0%	37.7		26.2	
			(4.5)	(6.7)	(2.2)	(1.3)	(5.0)		(15.8)	
4	S	459	18	15	3	8.1%	65	30	–	15
	E	449	16.8	18.2	3.6	8.7%	53.2	–	39	
			(14.3)	(3.1)	(2.7)	(3.9)	(11.5)		(34.0)	
5	S	498	69	147	6	44.6%	0	72	–	32
	E	498	71.8	44.5	1.3	23.1%	1.8	–	96	
			(7.4)	(68.4)	(1.0)	(13.2)	(0.6)		(69.6)	
6	S	430	4	63	3	16.3%	254	12	–	17
	E	430	1.6	64.4	1.6	16.5%	253		12.2	
			(0.5)	(2.2)	(1.4)	(0.4)	(2.0)		(0.5)	
7	S	743	29	221	3	34.1%	0	9	–	36
	E	743	12.8	24.8	1.3	7.3%	1.8		151	
			(12.3)	(17.0)	(0.5)	(2.2)	(1.7)		(95)	
8	S	497	33	183	13	46.1%	251	17	–	24
	E	492	24.6	88.4	1.6	31.8%	24.4		206	
			(25.0)	(47.7)	(7.2)	(12.2)	(25.3)		(54)	
9	S	310	3	0	0	1.6%	2	0	–	12
	E	312	1.6	1.8	1.6	1.7%	9.4		12.2	
			(1.1)	(1.3)	(3.9)	(1.3)	(11.1)		(13.2)	

*The mean numbers of AE according to the expert physicians are also reported with the SD between parenthesis. The breathing frequency f_breath_ (in breath per min) as evaluated by the SyncSmart software is also reported*.

*S, SyncSmart; E, experts; N_tot_, total number of detected ventilatory cycles in the automatic scoring (s) and the mean number of ventilatory cycles once the scoring is reviewed by the experts (E); IE, ineffective effort; AT, auto-triggering; DT, double-triggering; AI, asynchrony index; Bck, backup cycles; X, discarded from the analysis due to too high leakage; mod, modified by the expert; f_breath_, breathing frequency*.

**Table 3:** Sensitivity (Se), specificity (Sp), positive predictive value (PPV), κ-coefficient, and agreement for the automatic scoring by SyncSmart successively compared to each expert and for each expert successively compared to one another.

**AE**	**Sensitivity**		**Specificity**		**PPV**		κ**-coeff**.		**Agreement**	
	**Sync**.	**Inter**	**Sync**.	**Inter**	**Sync**.	**Inter**	**Sync**.	**Inter**	**Sync**.	**Inter**
N	**0.92**	0.90	0.84	0.78	0.91	0.90	0.37	0.33	0.87	0.83
	(0.10)	(0.10)	(0.21)	(0.25)	(0.10)	(0.10)	(0.13)	(0.13)	(0.14)	(0.13)
	*p* = 0.084		*p* = 0.23		*p* = 0.23		*p* = 0.13		*p* = 0.17	
Bck	0.63	0.60	**0.98**	0.96	0.64	0.62	0.30	0.28	0.74	0.72
	(0.45)	(0.40)	(0.03)	(0.05)	(0.45)	(0.41)	(0.22)	(0.19)	(0.34)	(0.26)
	*p* = 0.20		*p* = 0.0013		*p* = 0.15		*p* = 0.36		*p* = 0.10	
DT	**0.65**	0.55	**0.99**	0.99	0.62	0.56	0.29	0.26	0.75	0.72
	(0.40)	(0.41)	(0.02)	(0.03)	(0.37)	(0.41)	(0.18)	(0.19)	(0.27)	(0.26)
	*p* = 0.082		*p* = 0.0029		*p* = 0.26		*p* = 0.18		*p* = 0.47	
AT	**0.71**	0.60	**0.95**	0.97	0.62	0.60	**0.29**	0.26	0.77	0.75
	(0.38)	(0.36)	(0.10)	(0.08)	(0.40)	(0.36)	(0.19)	(0.16)	(0.24)	(0.18)
	*p* = 0.031		*p* < 10^−5^		*p* = 0.42		*p* = 0.0044		*p* = 0.35	
IE	**0.90**	0.81	**1.00**	1.00	**0.92**	0.81	**0.45**	0.40	**0.94**	0.88
	(0.21)	(0.16)	(0.01)	(0.01)	(0.20)	(0.17)	(0.10)	(0.18)	(0.15)	(0.21)
	*p* = 0.031		*p* = 0.046		*p* = 0.058		*p* = 0.0056		*p* = 0.030	

*SDs are reported in parenthesis*.

*PPV, positive predicitive value; Sync., SyncSmart vs. experts; Inter, an expert vs. another one; AE, asynchrony event; N, normal; Bck, backup cycle; DT, double-triggering; AT, auto-triggering; IE, ineffective effort*.

*Values in bold fonts are those for which SyncSmart has better results than the inter-rater variability*.

**Table 4:** Sensitivity (Se), specitify (Sp), PPV, κ-coefficient, and agreement between (i) the experts and the automatic scoring by SyncSmart and (ii) between experts, successively computed for each patient.

	**Sensitivity**		**Specificity**		**PPV**		**κ-coeff**.		**Agreement**	
	**Sync**.	**Inter**	**Sync**.	**Inter**	**Sync**.	**Inter**	**Sync**.	**Inter**	**Sync**.	**Inter**
P_1_	0.81	0.72	0.98	0.97	0.84	0.72	0.38	0.33	0.88	0.82
	(0.28)	(0.31)	(0.03)	(0.05)	(0.25)	(0.32)	(0.12)	(0.14)	(0.12)	(0.14)
P_2_	0.91	0.78	0.98	0.97	0.85	0.79	0.42	0.36	0.91	0.84
	(0.17)	(0.28)	(0.04)	(0.05)	(0.25)	(0.28)	(0.10)	(0.13)	(0.17)	(0.19)
P_3_	0.73	0.74	0.98	0.97	0.74	0.74	0.36	0.35	0.83	0.84
	(0.41)	(0.38)	(0.05)	(0.32)	(0.41)	(0.38)	(0.20)	(0.18)	(0.27)	(0.20)
P_4_	0.82	0.79	0.96	0.95	0.90	0.82	0.40	0.36	0.90	0.86
	(0.17)	(0.18)	(0.07)	(0.09)	(0.12)	(0.17)	(0.07)	(0.07)	(0.07)	(0.07)
P_5_	0.66	0.55	0.94	0.93	0.55	0.53	0.25	0.23	0.69	0.70
	(0.42)	(0.40)	(0.10)	(0.14)	(0.42)	(0.39)	(0.19)	(0.18)	(0.30)	(0.22)
P_6_	0.94	0.71	0.99	0.92	0.97	0.70	0.47	0.30	0.97	0.77
	(0.10)	(0.38)	(0.02)	(0.18)	(0.05)	(0.38)	(0.04)	(0.19)	(0.04)	(0.25)
P_7_	0.69	0.57	0.90	0.88	0.53	0.57	0.23	0.21	0.67	0.67
	(0.43)	(0.42)	(0.17)	(0.22)	(0.43)	(0.41)	(0.20)	(0.31)	(0.30)	(0.25)
P_8_	0.59	0.55	0.90	0.91	0.56	0.55	0.22	0.23	0.67	0.69
	(0.43)	(0.35)	(0.12)	(0.09)	(0.35)	(0.35)	(0.18)	(0.16)	(0.27)	(0.24)
P_9_	0.60	0.46	0.90	0.85	0.62	0.46	0.25	0.15	0.67	0.56
	(0.49)	(0.48)	(0.28)	(0.31)	(0.48)	(0.48)	(0.24)	(0.21)	(0.37)	(0.10)
$\bar{\text{P}}$	0.76	0.69	0.95	0.94	0.74	0.69	0.34	0.30	0.81	0.78
	(0.35)	(0.36)	(0.12)	(0.14)	(0.36)	0.36)	(0.18)	(0.17)	(0.25)	(0.22)

*SDs are reported in parenthesis*.

*Sync., SyncSmart; Inter, inter-scorer; PPV, positive predictive value*.

## 4. Discussion

### 4.1. Comparison With Other Studies

This study confirms that the inter-rater variability between physicians to detect PVA events during NIV by visual inspection is pretty large for the three main AEs (DT, AT, and IE). This was already shown by Longhini et al. (32). Most often physicians recognize correctly the presence of an AE but they had difficulties to discriminate them; for instance, it is rather difficult to distinguish DT from a combination of AT-N. Our results show that the events detected by automatic scoring were well recognized by expert physicians but scored as a different AE. This explains why the inter-rater variability is greater than the disagreement with the automatic scoring. This partly explains the large inter-rater variability for determining the type of PVA although the inter-rater variability in the AI is rather low (the variance is equal to 9.9%). For six of the nine patients, there is a slight trend in SyncSmart to miss some AEs (about 10% as detailed in Table 2) as exemplified with the AT shown in Figure 1 (*t* = 738 s) that is considered as a normal cycle. It is also known that IE may be missed from a simple waveform analysis (36). Conversely, in three patients (5, 7, and 8), there was an over detection of AT. Over- detection of AT occurred in two patients with ALS and one patient with COPD whose breathing frequencies were greater than 24 breath per min (see Table 2). Indeed, the other six patients had a breathing frequency that is lower and AT as correctly detected. Even with this overdetection, the main result of this study is that automatic scoring is within the inter-rater variability and provides, in general, a lower bound for the AI.

To our knowledge, this pilot study is the first attempt to the assess performance of software to automatically detect DT, AT, and IE from the pressure and flow waveforms. In most of the previous studies, only IEs were investigated (24, 25, 27, 31). These different algorithms were detecting IEs with Se and Sp greater than 0.90 as observed with SyncSmart. One study investigated DT in 67 patients but there is no information on the validation of the algorithm (29). Two studies investigated AE without distinguishing them. One reported the AI with a Se and a Sp at about 0.90 (28). The second one investigated the asynchrony between patient and ventilator (30). The latter study was, in fact, devoted to neurally adjusted non-invasive ventilator (NAVA), and the algorithm uses the diaphragm electrical activity, pressure, and flow waveforms. So, its purpose was different from our pilot study. None of these studies, whose main characteristics are reported in Table 5, investigated AT, which is, as revealed in this study, the most difficult AE to determine, even when the belt signal is provided.

**Table 5:** Brief overview of the studies devoted to automatic scoring of AE in mechanical ventilation.

**Reference**	**Events**	**Sensitivity**	**Specificity**		***N*_patient_**	***N*_breath_**
Mulqueenyl et al. (31)	IE	0.59	0.99	ICU-NIV	23	5,624
Chen et al. (24)	IE	0.93	0.93	ICU	14	5,899
Cuvelier et al. (25)	IE	0.95	1.00	NIV	9	2,127
Blanch et al. (27)	IE	0.92	0.80	ICU	8	1,024
Chiew et al. (28)	AI	0.90	0.88	ICU	11	5,701
de Haro et al. (29)	DT	—	—	ICU	67	9,694,573
Dorduin et al. (30)	AE	—	—	NIV	11	—

*The type of AE, sensitivity (Se), and specificity (Sp), when provided, the ventilation mode [intensive care unit (ICU) or non-invasive ventilation (NIV)], the number of patients analyzed, and the number of breaths are reported for each of them*.

*IE, ineffective effort; AI, asynchrony index; DT, double-triggering; AE, asynchrony event*.

In the study of Mulqueeny et al. (31), DTs were defined as two pressure cycles separated by less than 500 ms. This definition does not allow to distinguish a DT from a combination of AT-N. Distinguishing these two types of AEs is indeed sometimes very difficult, thus explaining the low Se found for these two types of AE, from the SyncSmart software as well as from the experts. In DT, the first oscillation of the flow has an amplitude that is larger than the second one (7, 37). During AT, the patient is still expiring: the inspired volume should be therefore smaller than for that normal cycle, thus inducing lower amplitude flow waveforms (7, 37). This is a possible way to discriminate DT from AT-N (37). It seems that when the breathing frequency is too high, such feature is no longer reliable: this could explain the over detection of AT observed in our study.

Some studies show that more than 30% of patients in NIV have more than 10% of AEs (11, 38, 39). In our cohort, 44% of patients had AI >30%. Omitting the over detected AT, 349 AEs were actually detected. This is not sufficient for robust results and investigating a larger cohort is required. Nevertheless, previous study showed that the detection of AEs is inversely related to their prevalence, indicating that the ability to recognize PVA is reduced when their occurrence increases (20, 32). For these reasons, asynchrony events were not too numerous during our ventilation sessions for optimizing the ability of physicians to correctly recognize the asynchrony events. The Se, PPV, κ-coefficients, and agreement are the lowest for the three patients, for which AT was over detected and, consequently, with AI>34%. Clearly, this drawback of SyncSmart should be corrected. As physicians are commonly doing for sleep scoring (40), in our study, experts start also from the automatic scoring.

### 4.2. Limitations of the Study

There are possible limitations in this study. The first being that the experts were not blind to automatic scores and had, in fact, to reviewed them. This may create a bias by reducing the inter-rater variability compared to scoring from raw tracings. Nevertheless, even with this bias, the statistics (κ-coefficients and agreement as reported in Table 3) are still in favor of automatic scoring since the expert scoring better matches with the automatic scoring than with each other. Indeed, the modified scores very often differ from one expert to the other. With blind scoring, the results becomes intractable as we observed in a preliminary study (non-published), leading us to adopt the procedure commonly used to assess the performance of automatic sleep scoring. As the aim of the present pilot study is to evaluate the SyncSmart software as a proof of concept, further study is warranted to evaluate it for longer NIV sessions and for a larger cohort of patients.

There is not yet a standardization for coding AEs for patients using NIV. There are few contributions in that direction which are based on visual inspection (6, 7, 41) and, consequently, which use qualitative arguments that may sometimes lead to subjective interpretation. Another problem is related to obstructions that are not considered in the present study. Two of the patients have a noticeable number of obstructions. These events were merged with backup cycles. In this study, as it is considered by the SyncSmart software, the ventilatory cycles are discarded (<5% of the number of breaths) from the analysis when leaks are too large: This avoids inappropriate tracings that could prevent physicians from correctly detecting PVA. The presence of high leakage is to be considered as a primary event during NIV, which needs to be corrected before any other action, adjustment of treatment, or settings that can be considered or recommended.

It should be noted that, in the daily clinical practice, physicians use “visual” signs as chest movement or signs of discomfort and possibly feedback provided by the patient. In addition to the waveforms (pressure, flow, and belt signal), the SyncSmart software provides the patient and ventilator frequencies, which are useful to identify PVA. These last two limitations are assumed to be a source for increasing the inter-rater variability (32). Since “minor” AEs as advanced and delayed cycling (pressure release) (42, 43) are not detected by the SyncSmart software, they were not considered in this study. As they are suspected to be important for inducing major AEs, detecting them would lead to a better understanding of the causes of the main events.

When the AI is typically greater than 10%, an adjustment of the ventilator settings is required (9, 11, 20). Since these adjustments are event type dependent, it is relevant to discriminate DT, AT, and IE. DT occurs to restrictive patient with a low breathing frequency (37). AT may contribute for 40% of all AEs (32). As pointed out by Longhini et al., “*because of the poor performance of visual inspection of ventilator waveforms, algorithms able to recognize patient-ventilator asynchrony might indeed represent an important advance for the management of patient underlying NIV*” (32). SyncSmart overcomes the lack of a tool for helping physicians to identify the source for poor mechanical coupling between patient and ventilator.

## 5. Conclusion

To our knowledge, this is the first study to show that it is possible to automatically detect AEs from solely pressure and flow waveforms and that the results are within the inter-rater variability. This pilot study shows that such a procedure can be used to validate automatic scoring of AEs. Most of the events from more than 4,200 ventilatory cycles were well-detected by the SyncSmart software. When the breathing frequency is lower than 24 breath per min, SyncSmart returns an AI slightly less than the at assessed by expert physicians. A validation with a larger cohort is required to evaluate whether the AI provided by automatic scoring could be considered as a lower bound.

## Data Availability Statement

The datasets analyzed for this study can be found in the web page http://www.atomosyd.net/spip.php?article222

## Ethics Statement

The studies involving human participants were reviewed and approved by Corporacio Parc Tauli (CIR2010/015). The patients/participants provided their written informed consent to participate in this study.

## Author Contributions

All the authors contributed to the literature search and to the study design. ML collected the data measured in the patients in the Pneumology Unit Care of the Corporacio Parc Tauli (Sabadell, Spain). All the authors, but CL, reviewed the automatic scoring provided by the software. CL processed the data provided by the software and by the manual review of the physician. The analysis of the data was performed by all the authors. The manuscript was prepared by CL and reviewed by all the other authors.

## Conflict of Interest

ML reports personal fees for lectures from Philips and Resmed. JA is part time employee at Hamilton Medical. He has received advisory honorarium from Breas Medical and ResMed. He has received honorarium for lecturing from Philips. AC has received fees for lecturing and roundtable meetings from Philips, Breas and Resmed. MC has received advisory honorarium from Breas Medical and ResMed. She has received honorarium for lecturing from, Philips, ResMed, Breas and MPR. CL has received fees for consulting from Breas Medical. JS reports grants and personal fees for lectures from Heinen und Löwenstein and VitalAire, grants, personal fees for lectures and non-financial support for meeting attendance from Vivisol GmbH, grants from Weinmann Deutschland, personal fees for consultancy/advisory board work from Breas Medical, during the conduct of the study; personal fees for consultancy and lectures, and non-financial support for meeting attendance from Boehringer Ingelheim Pharma, personal fees for consultancy and lectures from SenTec AG, Keller Medical GmbH, Linde Deutschland and Santis GmbH, outside the submitted work. NH has received unrestricted research grants from Philips, Philips-Respironics, B&D Electromedical, Breas and Fisher-Paykel. He has has received fees for lecturing and roundtable meetings from Philips, Philips-Respironics, B&D Electromedical, Breas and Fisher-Paykel. SN has received fees for lecturing and roundtable meetings from Philips, Breas and Resmed. He is on the advisory board for Breas and Philips. The remaining authors declare that the research was conducted in the absence of any commercial or financial relationships that could be construed as a potential conflict of interest.
